# Provident Dispensaries and the Bill

**Published:** 1911-07-08

**Authors:** 


### PROVIDENT DISPENSARIES AND THE BILL.

A CONFERENCE of representatives of dispensaries in London and the Provinces was held in London on June 28. Letters were read from dispensaries in many parts of the country. The feeling was unanimous that the National Insurance Bill as it stood would be most prejudicial to dispensaries whether they were provident or free, and in many cases would practically destroy them. It was pointed out that in some towns as many as a third or more of the population were members of provident dispensaries. A committee was appointed to communicate with dispensaries throughout the country, with a view to their concerting measures for submitting their case to the Chancellor of the Exchequer. The following resolution was passed unanimously: "That in the interest of many hundred thousands of the working classes who receive medical aid from dispensaries all over the country it is most desirable that these institutions, many of which have existed for very many years and are approved by the medical profession, should be utilised for the administration of medical benefits under a national insurance scheme."

